# Evaluation of some immune and inflammatory responses in diabetes and HIV co-morbidity

**DOI:** 10.4314/ahs.v23i1.14

**Published:** 2023-03

**Authors:** Rebecca Chinyelu Chukwuanukwu, Ngozi Bernice Nwosu, Martin Ositadinma Ifeanyichukwu, Augusta Chinyere Nsonwu-Anyanwu, Patrick Onochie Manafa

**Affiliations:** 1 Medical Laboratory Science Department, Nnamdi Azikiwe University, Nnewi Campus, Nigeria; 2 Medical Laboratory Science Department, University of Calabar, Calabar, Nigeria

**Keywords:** Diabetes, HIV, co-morbidity, immune responses, inflammation, glycemic control

## Abstract

**Background:**

Co-existence of diabetes in the HIV infected reportedly further complicates the attendant impairment of immunity and increases susceptibility to opportunistic infections.

**Objective:**

This study aimed to evaluate some immune and inflammatory parameters in HIV and type 2 diabetes (T2D) co-morbidity: Immunoglobulin M and G (IgM and IgG), Interleukin-6, CD4+ T-cells and C-reactive protein.

**Method:**

The study involved 200 subjects grouped according to their HIV and diabetes status: Group 1 ‘Diabetic HIV seropositive’ (n=40), Group 2 ‘Non diabetic HIV seropositive’(n=60), Group 3 ‘Diabetic HIV seronegative’(n=50), and Group 4 'Control non diabetic HIV seronegative'(n=50). Blood samples were collected for testing.

**Results:**

CRP levels were significantly elevated in diabetes and HIV co-morbidity compared to other groups. IL–6 levels were significantly higher in diabetics with or without HIV infection. In addition, IL-6 was significantly elevated in individuals with poor glycemic control (HbA1c > 9.0%) compared to those with good glycemic control. IgG and IgM levels in diabetic HIV seropositive subjects were highest compared with other groups.

**Conclusion:**

The increased IL-6, CRP, IgG, IgM and decreased CD4+ T cell counts observed in co-morbidity suggest that HIV and T2D co-morbidity exacerbate the immune and inflammatory impairment observed in either disease entity.

## Introduction

Diabetes morbidity has reportedly increased in the past two decades at a disturbing rate worldwide, and is now one of the leading causes of death in low-middle income countries^1^. Of greater concern is the interaction between diabetes and HIV which is prevalent in sub-Saharan Africa. In sub-Saharan Africa, there is an increasing prevalence of diabetes and hypertension among adults and this is in a region with a large population of people living with HIV/AIDS (PLWH) who are on anti-retroviral therapy^2^. Indeed, there have been reports of growing burden of cardiometabolic diseases among PLWH^3^,^4^,^5^. Earlier reports link the development of type 2 diabetes (T2D) with certain anti-retrovirals (ARVs)^6^,^7^,^8^. HIV infection and certain ARVs are known to be associated with development of insulin resistance and diabetes^9^,^1^. Nansseu et al^10^ report a rapidly increasing burden of diabetes and pre-diabetes among ART exposed HIV infected patients. Though the mortality due to HIV has significantly improved, patients with co-morbid diabetes have a much greater challenge^9^. As a result of increased risk of developing T2D, international guidelines recommended yearly screening of all HIV-infected individuals for diabetes^11^,^12^,^13^.

Chronic immune activation and inflammation are consistently found in PLWH and T2D is also characterized by similar findings^14^. Low grade systemic inflammation has been hypothesized as an underlying factor for the pathogenesis of T2D^15^,^16^, with elevated plasma levels of C-reactive protein (CRP) and IL-6^16^. CRP, an acute phase protein is synthesized by hepatocytes in response to pro-inflammatory cytokines, in particular IL-6^17^. Ingle and Patel^18^ reported that hyperglycaemia stimulates the release of the inflammatory cytokines, tumour necrosis factor (TNF-α) and IL-6 from various cell types and can also result in the induction and secretion of acute phase reactants by adipocytes. CRP is an acute phase protein elevated in HIV infection. In HIV infection, Immunoglobulin G (IgG) and Immunoglobulin M (IgM) concentrations increases as disease progresses while in diabetes, non-enzymatic glycation of proteins and other bio-molecules have been implicated to be responsible for failure of humoral immunity in diabetic patients^19^.

Some studies observed genetic, socioeconomic difference in the incidence rate of diseases associated with inflammatory response^20^,^21^,^22^. Hadigan and Kattakuzhy reported limited data on the impact of ethnicity and race on diabetes in HIV setting^23^.

In view of these, this study was designed to seek a better understanding of the immune and inflammatory responses in HIV and T2D co-morbidity in this population. Thus, this study assessed the dual effect of T2D and HIV co-morbidity in this population with a relatively high burden of people living with HIV and increasing incidence of T2D.

## Materials and methods

This case control study was carried out at St. Charles Borromeo Specialist Hospital, Onitsha. Informed consent was obtained from all study participants. Ethical approval for the research design was obtained from the institutional review board for research ethics of the St. Charles Borromeo Hospital, Onitsha and Faculty of Health Sciences and Technology, Nnamdi Azikiwe University Nnewi Campus, South-East, Nigeria. The conduct of the study was in compliance with the ethical principles guiding medical research involving human subjects as declared in Helsinki in 1975 and subsequent revisions of the declaration. Informed consent was sought and obtained from all study participants.

### Subjects

A total of two hundred subjects comprising type 2 diabetics and HIV seropositive individuals and their respective controls were recruited from the St. Charles Borromeo Specialist Hospital Onitsha in South-East, Nigeria. The subjects were grouped according to HIV and diabetes status as: Group 1: ‘Diabetic HIV seropositive subjects’ (n = 40); Group 2: ‘Non diabetic HIV seropositive’ (n = 60); group 3: ‘Diabetic HIV seronegative’ (n = 50); and Group 4: Control ‘Non diabetic and HIV seronegative’ (n = 50). According to the CDC and WHO HIV disease staging, only HIV Stage II subjects were recruited and had commenced on anti-retroviral therapy. Blood samples were collected for assessing fasting plasma glucose (FPG), glycated haemoglobin (HbA1c), CD4^+^ T cell counts, C-reactive protein (CRP), Interleukin - 6 (IL-6), Immunoglobulin M (IgM) and Immunoglobulin G (IgG) using standard methods as will be further described.

### Sample collection

The samples used for the study were whole blood, plasma and serum. These were obtained as follows: Seven millilitres (7mls) of whole blood were collected from each participant, and 2ml dispensed into fluoride oxalate tube, 2ml into EDTA tube, and 3ml into plain tube. Plasma and serum were separated according to standard operating procedure and the FBS assayed within 2 hours. Serum for other analyses were stored frozen at -70oC until the required number of batched samples for assay were obtained. CD4 + T cell count and glycated haemoglobin level were assayed from the EDTA whole blood within 4 hours of sample collection.

### Laboratory methods

According to national serial algorithm, participants were tested for HIV-1 and HIV-2 using two different immunochromatographic methods: Determine 1 & 2 (Inverness Medical Japan Co, Ltd) and STAT-PAK (Chembio Diagnostic System, New York, USA). In the case of a tie, a third method (Unigold) was used to break the tie. Tests were carried out according to manufacturer's instructions. CD4^+^ T cells were counted using the Cyflow SL.3 cytometer counter (Partec Cyflow® Germany). This uses a green solid-state laser with an excitation light source of 532nm. Partec test kits used to prepare the samples contain mouse monoclonal antibody (mAb) isotype IgG1 clone MEM-241 which recognizes the human CD4 antigen.

IgM and IgG were determined by Immunoturbidimetric measurement (Cromatest Linear Chemical, S.L Barcelona Spain). Anti- human IgG and IgM antibodies form insoluble immune complexes when mixed with samples containing IgG or IgM. The scattering light of the immune complexes depends on the IgG or IgM concentration in the patient sample and can be quantified by comparison from a calibrator of known IgG and IgM concentration. Protocols were followed according to manufacturers' instruction. Colour developed were read spectrophotometrically at 540 nm wavelength and absorbance was recorded. IgG and IgM in the sample was calculated by interpolation of its absorbance value using the calibration curve.

CRP was assayed by Enzyme Linked Immunosorbent Assay (Perlong, BioTechnology Company Ltd. Beijing China). The assay was carried out according to manufacturer's instruction. The absorbance of each well was read in a microplate reader at 450 nm wavelength.

IL-6 levels were assayed by ELISA (Bisino Bio Technology and Science Inc. Beijing China), and protocol was as per manufacturer's instructions. The absorbance of each well was read at 450 nm. The concentration of IL-6 in the samples were then read from a standard curve plotted from the assay of known standard.

Fasting plasma glucose was estimated by the glucose oxidase method (ELITech Clinical Systems SAS, France). Glucose was determined after enzymatic oxidation in the presence of glucose oxidase. The hydrogen peroxide formed reacts, under catalysis of peroxidase, with phenol and 4 – aminophenazone to form a red – violet quinoneimine dye as indicator, the colour intensity is proportional to the concentration of glucose in the sample. The absorbance of the coloured solution was read at 546nm within 60 minutes.

Glycated haemoglobin (HbA1c) was estimated by non-enzymatic cation-exchange resin method (using commercial reagent kit by Teco Diagnostics, USA). This method employs a weak binding cation-exchange resin for rapid separation of glycohaemoglobin (fast fraction) from non - glycosylated haemoglobin.

### Statistical analysis

Data were analysed and comparisons performed using mean, standard deviation, median, student t- test, Mann-Whitney, ANOVA and Pearson's correlation (SPSS version 22 and Graph Pad Prism 8). Level of significance was set at P< 0.05. Sample size was estimated using the formula by Naing et al, 2006 [24] with national HIV prevalence of 3.4% and diabetes prevalence of 2.4%.

## Results

A total of 200 subjects were studied to assess the effect of diabetes and HIV co-infection on the immune and inflammatory responses. The age and sex of the recruited subjects in the various groups are shown in Table 1.

**Table 1:** Demographic characteristics of the study population

Groups	Age range	Sex
Diabetic HIV seropositive subjects (n=40)	33–55	Male:20 Female:20
Non diabetic HIV seropositive subjects (n=60)	34–48	Male:29 Female:31
Diabetic HIV seronegative subjects (n=50)	37–53	Male:23 Female:27
Non diabetic HIV seronegative (n=50)	35–49	Male:28 Female:22
ANOVA	0.100	0.309

Level of significance set at P<0.05.

Table 2 shows the CD4^+^ T cell counts, IL-6 and CRP in the study groups. There were significant differences in the CD4^+^ T cell counts. Lower CD4^+^ T cell counts in the diabetic HIV seropositive group compared to the diabetic HIV seronegative group (P<0.001) and non diabetic HIV seronegative group (control group) (P<0.001) see table 2. There was no significant difference between the diabetic HIV seropositive group compared to non diabetic HIV seropositive groups. There was significantly lower CD4^+^ T cell count in non diabetic HIV seropositive group compared to diabetic HIV seronegative group (P=0.002). Interestingly, there was a significantly lower CD4^+^ T cell count in diabetic HIV seronegative group compared to non diabetic HIV seronegative group (P=0.015).

**Table 2:** Mean (+/-S. D) values of CD4+, Median (IQR) of IL – 6 and CRP in the various groups

Groups	CD4^+^ (cells/mm^3^) Mean (± SD)	IL-6 (ng/l) Median (IQR)	CRP (mg/l) Median (IQR)
Diabetic HIV seropositive subjects (1) (n=40)	427 ± 218	8.7 (5.1–13.5)	3.7 (1.9–5.2)
Non diabetic HIV seropositive subjects (2) (n=60)	490 ± 230	5.6 (3.4–7.1)	2.3 (1.2–4.7)
Diabetic HIV seronegative subjects (3) (n=50)	817 ± 209	6.9 (2.2–8.8)	0.9 (0.6–1.8)
Non diabetic HIV seronegative (4) (n=50)	866 ± 185	5.0 (1.9-6.5)	0.2(0.1–0.3)
ANOVA	**< 0.001**	**0.002**	**< 0.001**
1 vs 2	0.1710	**0.015**	**0.02**
1 vs 3	**< 0.001**	0.412	**<0.001**
1 vs 4	**<0.001**	**0.004**	**<0.001**
2 vs 3	**<0.001**	0.221	**<0.001**
2 vs 4	**<0.001**	**0.004**	**<0.001**
3 vs 4	**0.015**	**0.009**	**<0.001**

KEY : n = number, 1: Diabetic HIV seropositive subjects, 2: Non diabetic HIV seropositive subjects, Group 3: Diabetic HIV seronegative subjects, Group 4: Non diabetic HIV seronegative (control) subject. CD4+=CD4+ T cells, IL-6=Interleukin 6, CRP=C-reactive protein. Level of significance set at P<0.05. Significant values in bold. *Some non-significant permutations not shown.

The results of the IL-6 reveal statistically higher IL-6 levels in the diabetic HIV seropositive group compared to non diabetic HIV seropositive group (P=0.015) and the control non diabetic HIV seronegative group(P=0.004) (See table 2), while there was no significant difference with the diabetic HIV seronegative subjects (P=0.412). Also, there was no significant difference between the non-diabetic HIV seronegative group and the diabetic HIV seronegative group (P=0.221).

The findings for the CRP are also shown in table 2. There were significant differences when comparing CRP between all groups.

Table 3 shows the fasting plasma glucose (FPG) levels in the study population. As expected, FBS levels were significantly different in the diabetic groups compared to the non diabetic groups. Notably, the FPG level was significantly higher in non diabetic HIV seropositive subjects compared to non diabetic HIV seronegative group (P=0.012) (See table 3).

**Table 3:** Mean ± SD values of FBS, HbA1c, IgM, and IgG in the various study groups

Groups	FPG (mmol/l) Mean (+SD)	HbA1c (%) Mean (+SD)	IgM (mg/dl) Mean (+SD)	IgG (mg/dl) Mean (+SD)
Diabetic HIV seropositive subjects (1) (n=40)	9.1 ± 3.3	10.0 ± 3.6	134.6 ± 19.6	1549.8 ± 205.9
Non diabetic HIV seropositive subjects (2) (n=60)	4.7 ± 0.5	4.7 ± 0.4	125.1 ± 15.3	1531.2 ± 205.6
Diabetic HIV seronegative subjects (3) (n=50)	9.9 ± 4.6	8.8 ± 3.2	106.0 ± 14.4	1495.6 ± 291.2
Non diabetic HIV seronegative (4) (n=50)	4.4 ± 0.4	4.5 ± 0.4	76.3 ± 9.8	731.4 ± 127.9
ANOVA	**<0.001**	**<0.001**	**<0.001**	**<0.001**
1 vs 2	**<0.001**	**<0.001**	**0.008**	0.659
1 vs 3	0.363	0.085	**<0.001**	0.323
1 vs 4	**<0.001**	**<0.001**	**<0.001**	**<0.001**
2 vs 3	**<0.001**	**<0.001**	**<0.001**	0.4546
2 vs 4	**0.012**	**0.004**	**<0.001**	**<0.001**
3 vs 4	**<0.001**	**<0.001**	**<0.001**	**<0.001**

KEY: = significant at p<0.05, n = sample size, Group 1 = Diabetic HIV seropositive subjects, Group 2 = Non diabetic HIV seropositive subjects, Group 3 = Diabetic HIV seronegative subjects, Group 4 = Non diabetic HIV seronegative (control) subject. Significant values in bold.

The result for glycated haemoglobin mirrored the results of FPG. A significant difference was also seen as well between non diabetic HIV seropositive and non diabetic HIV seronegative (P=0.004).

There were significant differences in all the groups for IgM. However, for IgG, the only significant difference seen was when comparing each study group with the control non diabetic HIV seronegative group (Table 3).

Table 4 shows findings when comparing the study parameters in patients who had good glycemic control (HbA1c <9.0%) and those who had poor glycemic control. There were significantly higher IgG levels in those with good glycemic control compared to those with poor glycemic control (table 4) suggesting better immunoglobulin responses in good glycemic control (P=0.039).

**Table 4:** Effect of glycemic control on parameters studied. The mean ± SD in good glycemic control (HbA1c <9.0%) and poor glycemic control (HbA1c >9.0%) among diabetic HIV seropositive group

PARAMETERS	Good glycemic control (<9.0 %) n = 21	Poor glycemic control (>9.0 %) n = 21	P - VALUES
CD4^+^ (cells/mm^3^)	442.0 ± 237.2	414.0 ± 203.7	0.725
FBS (mmol/l)	8.4 ± 2.8	9.8 ± 3.7	0.315
IgG (mg/dl)	1556.4 ± 167.2	1342.5 ± 246.3	**0.039**
IgM (mg/dl)	138.9 ± 17.1	129.7 ± 21.3	0.471
IL – 6 (ng/l)	7.7(2.2–11.2)	12.6(6.2–13.5)	**0.002**
CRP (mg/l)	4.1(0.9–2.2)	3.8(1.3–3.2)	0.084

KEY: n = sample size, * = significant at p< 0.05. Significant values in bold.

IL-6 levels were significantly higher in poor glycemic control compared to good glycemic control (P=0.002).

There was a negative correlation between CD4 and CRP (r = -0.289, p=0.014) amongst males while among females, there was also negative correlation (r = 0.330, p= 0.003). Among the female subjects, CD4 show a positive correlation with IL-6 (r = 0.238, p= 0.036) (not shown). There were significantly higher IL-6 levels among females compared to males in the diabetic HIV seropositive group (P=0.022) (not shown). However, levels of IL-6 were higher in males in the non diabetic HIV seropositive group.

## Discussion

Among the study population, mean CD4^+^ T cell count was lowest in the co-morbid diabetic HIV seropositive group of subjects. This suggests that the interaction between diabetes and HIV exacerbates the depletion of CD4+ T cells seen in HIV infected subjects. It is interesting to note that there was a significantly lower CD4+ T cell counts in diabetic HIV seronegative group compared to non diabetic HIV seronegative group (P=0.015) in this population, suggesting that T2D could deplete CD4+ T cells. Further studies with a larger number of T2D participants would be required to assess this finding in this population.

Notably, FPG levels were significantly higher in the non diabetic HIV seropositive compared to non diabetic HIV seronegative group (P=0.012). This is mirrored by findings for HBA1c with significantly higher levels in non diabetic HIV seropositive compared to non diabetic HIV seronegative group (P=0.004). This is indicative that HIV is linked to development of T2D. There is a possibility that this could be due to the HIV disease itself or due to drugs used to treat the disease as previously reported by Rhee et al 1 or perhaps even both. HIV infection itself is associated with increased risk of insulin resistance, while ART is associated with metabolic derangement and the occurrence of T2D 25. Various HAART have been shown to increase insulin resistance and reduce insulin secretion by interfering with glucose transporter system 6. A systematic review by Nansseu et al 10 reported that major risk factors for diabetes and prediabetes include Black or Hispanic origin, increased baseline fasting glycemia and certain ART regimens among others. Findings from this study show that HIV is associated with increased fasting plasma glucose and glycated haemoglobin levels in this population.

This study observed increased IgM and IgG in the HIV infected subjects with or without diabetes. The observed increase of IgM and IgG suggest the participation of these classes of immunoglobulin in protective immunity to HIV infection 26. However, the Immunoglobulin levels (both IgG and IgM) were highest in the diabetic HIV seropositive group, that is, in co-morbidity compared to all other groups. This suggests a synergistic effect on immunoglobulin levels. This finding could be similar to observations made by Odhiambo et al who reported that HIV infection drives IgM and IgG3 subclass bias in *Plasmodium falciparum*-specific and total immunoglobulin concentration^27^.

Both diabetes and HIV drive elevated levels of C-reactive protein as seen from our results, however, IL-6 could be better marker than CRP for the inflammation seen with diabetes and even in HIV co-morbidity. This agrees with findings by Hundhausen et al who though they worked on Type 1 diabetes, reported enhanced IL-6 responsiveness in diabetes 28. IL-6 is a multifunctional cytokine with a role in chronic inflammation 29,28. In addition, it was observed that IL-6 levels were significantly more elevated in diabetics with poor diabetic control when compared to their counterparts with good diabetic control.

Findings from this study revealed sex-related differences in IL-6 levels, with IL-6 being significantly higher in the female subjects with co-morbidity (diabetic HIV seropositive) compared to their male counterpart. Conversely, in the non diabetic HIV seropositive group, the males had significantly higher levels of IL-6 compared to the females. This has been previously reported by Akase et al 30. On the other hand, in the diabetic HIV seronegative group, the trend reverses with female participants having higher levels of IL-6 compared to their male counterparts. This is in line with the study by Panagi et al 31. They observed different inflammatory stress response pathways in men and women with Type 2 diabetes, with women producing increased plasma IL-6 compared to men. They suggested that long-term effects of these response patterns upon health need to be determined in future studies. Some previous studies have reported that circulating IL-6 levels do not vary significantly between the sexes 32,33,34. It has been speculated that gender-related difference exists in the control of IL-6 levels due to differences in dehydroepiandrosterone (DHEA) between male and female^34^. Findings from this study show that this was the case only in the non diabetic HIV seronegative group, that is, the apparently healthy control group, where there were no significant differences based on gender. Our findings point to the synergistic effect of diabetic HIV co-morbidity on hormonal influence in IL-6 levels. Interestingly, the difference in IL-6 in the sexes is higher in diabetic HIV seropositive group and diabetic HIV seronegative group compared to the non diabetic HIV seropositive group. This suggests that diabetes drives higher IL-6 levels.

## Conclusion

Our results show that HIV, T2DM or co-morbidity is associated with significantly lower CD4+ T cell counts. Immunoglobulin levels are higher in co-morbidity suggesting no adverse effect of co-morbidity on antibody responses. Increased inflammation as seen by IL - 6 and CRP levels were observed in co-morbidity suggesting that diabetes and HIV co-morbidity worsen the inflammation observed in either disease entity. IL-6 could be better marker than CRP for the inflammation seen with diabetes and even in co-morbidity, more so as IL-6 could differentiate good from poor glycemic control. This could be exploited for studies on T2D disease control and monitoring treatment success.

## Data Availability

Data are available upon request and may be obtained by contacting the corresponding author

## Supplementary Materials

Supplementary data table 1: Pearson correlations coefficient values of studied parameters based on gender among diabetic HIV seropositive, non diabetic HIV seropositive and diabetic HIV seronegative subjects.
